# CT-guided microwave ablation through the lungs for treating liver tumors near the diaphragm

**DOI:** 10.18632/oncotarget.17422

**Published:** 2017-04-26

**Authors:** Han Qi, Hao Zhang, Chao Wan, Lin Xie, Ze Song, Weijun Fan

**Affiliations:** ^1^ Department of Imaging and Interventional Radiology, Sun Yat-sen University Cancer Center, State Key Laboratory of Oncology in South China, Collaborative Innovation Center of Cancer Medicine, Guangzhou, China; ^2^ Department of Interventional Medical Center, The Affiliated Hospital of Qingdao University, Qingdao, China

**Keywords:** CT-guidance, microwave ablation, liver tumor, diaphragm, treatment

## Abstract

**Purpose:**

To explore the short-term efficacy and safety of CT-guided microwave ablation (MWA) for treating liver tumors near the diaphragm.

**Results:**

The complete response (CR) rate for CT-guided MWA through the lung was 94.7% (124/131). The incomplete response (ICR) rate was 5.3% (7/131), of which 6 patients with ICRs achieved CRs after MWA. The CR rate for Group I was higher than Group II (99.0% vs. 80.0%, *P=0.001*). The mean follow-up time was 11.2 ±7.50 months. The total local recurrence (LR) rate was 15.3% (20/131). The complication rate was 26.5%, and no severe complications were recorded. All complications were controllable and treatable. The incidence of diaphragmatic thickening during the MWA was 18.8% (*P*>0.05); the incidence of exudative changes inside the lungs was 6.8% (*P*>0.05).

**Conclusions:**

CT-guided MWA can detect changes in liver tissue, in the diaphragm and nearby lung tissues during the ablation process. It’s safe and effective to treat tumors close to the diaphragm by CT-guided MWA through the lung.

**Methods:**

CT-guided MWA was used on 131 tumors that were close to the diaphragm (distance between tumor and diaphragm ≤ 5 mm) in 117 patients with liver cancer. The tumors were divided into a < 3.0 cm group (Group I, n= 101) and a ≥ 3.0 cm group (Group II, n= 30) based on tumor diameters. The complications within 2 weeks following treatment were counted, and the safety and short-term efficacy of MWA were analyzed.

## INTRODUCTION

Image-guided thermal ablation therapy has become the standard treatment for small liver tumors [1–4]. Ultrasound-guided percutaneous microwave ablation (MWA) has been widely applied for liver tumor treatment due to ease of operation and real-time monitoring; [5–8] however, because ultrasound is usually impacted by pulmonary gas and ribs, it is hard to have a clear display for liver tumors close to the diaphragm [9–12]. Artificial pleural effusion and artificial ascites help to solve the problem in part, but both techniques have their own limitations. For tumors close to the diaphragm, CT imaging has high resolution and accurate positioning [13, 14]. Thus, CT-guided MWA can optimize the needle track for the best effect. It is often necessary to puncture through the lung tissues to reach the liver tumors accurately. The therapeutic efficacy of MWA through lung tissues for treating liver tumors close to the diaphragm is a matter of debate. In addition, whether or not there are more complications and whether or not the size of the tumors affects the MWA results and complication rate have not been established. Currently, there is no literature on these perspectives. To evaluate the short-term efficacy and safety of CT-guided MWA, we retrospectively studied 131 liver tumors close to the diaphragm in 117 patients.

## RESULTS

### Therapeutic efficacy

CT-guided MWA through the lung was successfully carried out on all 131 liver tumors close to the diaphragm in 117 patients. The average MWA power was 60.4±6.8 watt (range: 50-80 watt), and the average MWA time was 9.5±3.3 min (range:3-30min). The CR rate 1 month after MWA was 94.7% (124/131). The ICR rate was 5.3% (7/131), of which 6 patients with residual lesions achieved CR after complementary MWA. One patient had an active residue at the lesion edge, but declined further treatment, and the tumor shrank in the subsequent follow-up. The CR rates for Groups I and II were 99.0% (100/101) and 80.0% (24/30), respectively. The CR rate for Group I was greater than Group II (χ2=16.525, P=0.001).

The total LR rate was 15.3% (20/131). The LR rate for Groups I and II were 12.9% (13/101) and 23.3% (7/30), respectively; there was no statistical difference between the two group (χ2=1.957, P=0.245). The median follow-up time was 11.0 months (average, 11.2 months ± 7.49; range, 1-36 months).

### Complications

The total complication rate was 23.6% (31/131), including 20 patients with mild right-sided pneumothoraces (15.3%), 6 patients with mild right-sided pleural effusions (4.6%), 3 patients with mild hepatic subscapular bleeding (2.3%) and 2 patients with mild pulmonary bleeding (1.5%). The total complication rate for Group II was higher than group I (43.3% vs. 19.8%, *χ*^*2*^= 6.796, *P*= 0.015; Table 1). In subgroup analysis, the incidence of pneumothoraces of Group II was also higher than Group I. There were no treatment-related deaths or disabilities.

**Table 1:** Grouped complications according to tumor size

Complications	Grouped According to Tumor Size		*χ2*	*P*
	Group I (101)	Group II (30)		
**Mild pulmonary bleeding**	2	-		
**Mild right pneumothorax**	12	8	3.909	0.048
**Mild right pleural effusion**	4	2	0.388	0.620
**Mild hepatic subscapular bleeding**	2	1	0.189	1.000
**Total**	**20**	**11**	3.642	0.085

The tumors were sorted to Group I if the size of the tumor was < 3.0 cm and the tumors were sorted to Group II if the size of the tumor was ≥ 3.0 cm.

### Stress response

There were 22 patients (18.8%) with diaphragmatic thickening occurred during MWA. There were 8 patients (6.8%) with pulmonary exudative changes. With respect to Groups I and II, the diaphragmatic thickening rates were 13.9% and 26.7% (*χ2*=2.714, *P*=0.161), respectively, and the pulmonary exudation rates were 5.0% and 10.0% (*χ2*=1.029, *P*=0.383), respectively.

## DISCUSSION

**Figure 1:**
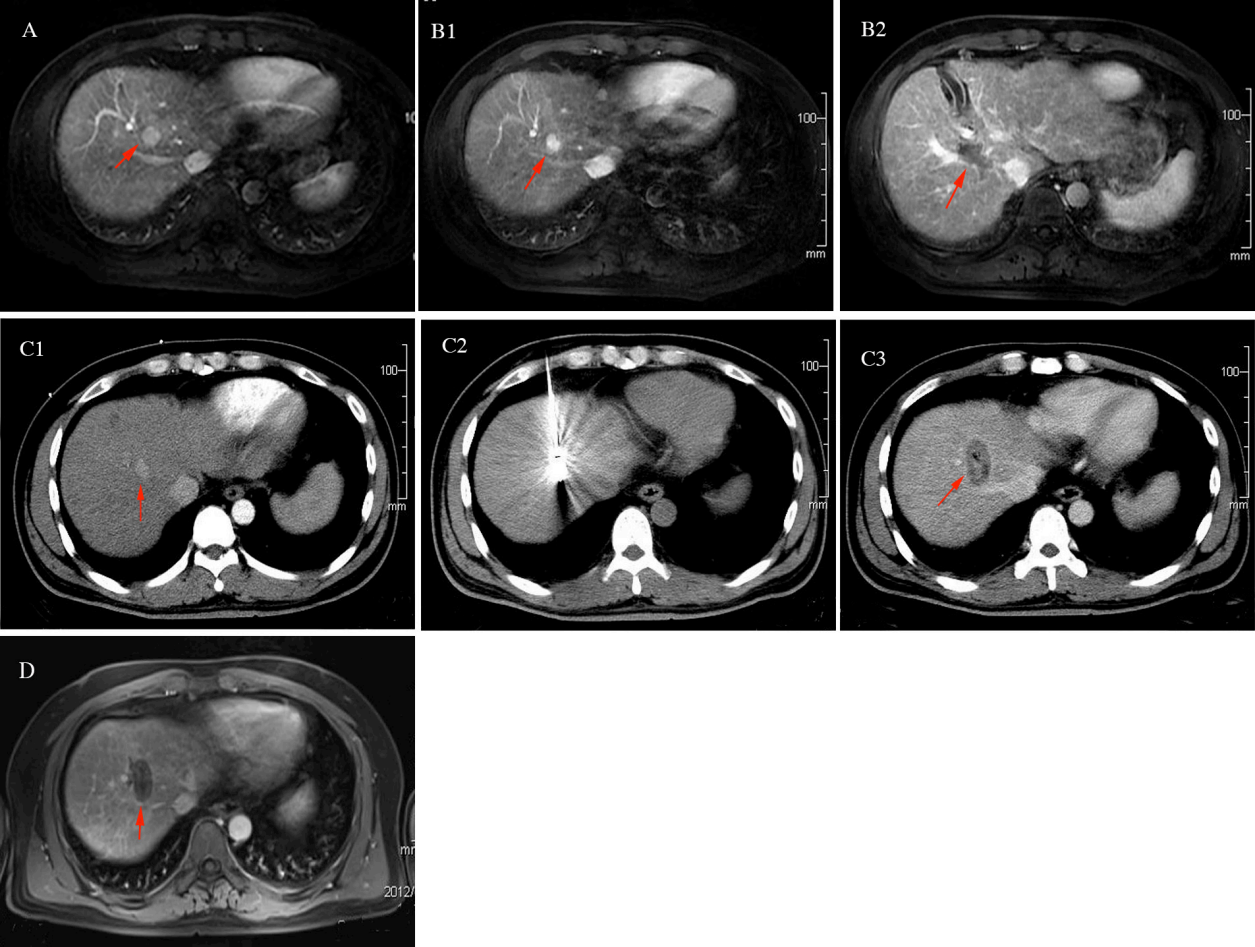
A 68-years-old male patient with primary liver S8 cancer underwent ultrasound-guided microwave ablation previously, but off-target effect appeared one month postoperatively The patients thus underwent CT-guided microwave ablation (MWA) in our hospital. There was no indication of tumor residue or recurrence in the follow-ups. **(A)** The upper abdomen MRI indicated: liver S8 tumor with the diameter of 1.2 cm (red arrow); **(B)** Off-target phenomenon occurred after ultrasound-guided MWA: The upper abdomen MRI review: the liver S8 lesion was still there without any significant changes (B1, red arrow), but there was a type of round shaped and low signal area (B2, red arrow) beside the lesion. **(C)** CT-guided MWA for liver tumor near the diaphragm: C1: Contrast-enhanced CT clearly manifested the liver S8 lesion (red arrow) before MWA. C2: MWA antenna precisely inserted into the tumor during the ablation process, with its needle tip slightly exceeded the edge of the tumor. C3: Contrast-enhanced CT immediately after MWA: ablation area completely covered the tumor (red arrow), without tumor residue. **(D)** The upper abdomen MRI review one month postoperatively: tumor achieved complete response without residue.

**Figure 2:**
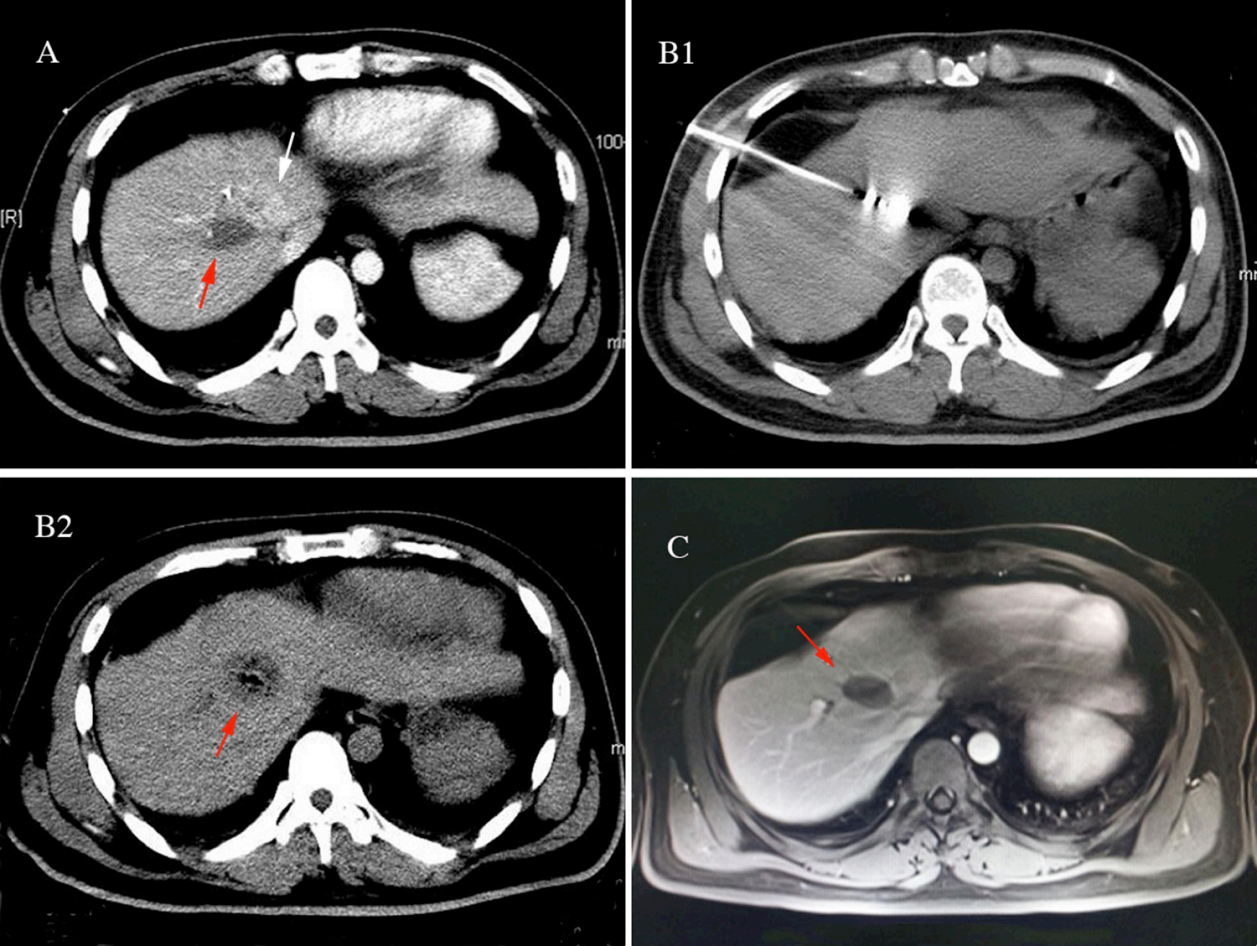
A 57-year-old female found a liver S8 metastatic tumor with diameter of 1.8cm 2 years after modified radical mastectomy of the left-side infiltrating ductal carcinoma received the ultrasound guided MWA The postoperative review implied only a small portion of lesion was ablated, the patients experienced the partially off-target effect. The patients then underwent CT-guided microwave ablation (MWA) in our hospital. There was also no indication of tumor residue or recurrence in the follow-ups. **(A)** Partially off-target phenomenon occurred after ultrasound-guided MWA: The contrast-enhanced CT review: a small portion of the liver S8 lesion was ablated (white arrow), but there was a low-density area (red arrow) beside the lesion. **(B)** CT-guided MWA for liver tumor near the diaphragm: B1: MWA antenna precisely inserted into the tumor during the ablation process, with its needle tip slightly exceeded the edge of the tumor. B2: Contrast-enhanced CT immediately after MWA: ablation area completely covered the tumor (red arrow), without tumor residue. **(C)** The upper abdomen MRI review 2 months postoperatively: tumor achieved complete response without residue (red arrow).

With the development of image-guided thermal ablation technology, MWA and radiofrequency ablation (RFA) have been increasingly used to treat liver cancers [1–4]. The short- and long-term therapeutic efficacy of thermal ablation is comparable to surgery in liver cancer [15]. Compared to RFA, MWA has unique advantages. Specifically, MWA has the following advantages: a fast heat generation, high intratumoral temperature, short ablation time and large ablation range. MWA is an open system which does not need an extracorporeal electrode plate. Furthermore, MWA has a strong ability to penetrate tissues with high frequency and the effect is synergistic combined with multiple antennas. MWA is less affected by carbonization and blood perfusion flow. In contrast, RFA is a closed system that requires an extracorporeal electrode plate to form a closed-loop, leading to a low intra-tumoral temperature, a long ablation time, and a small ablation range. Compared to RFA, the single antenna MWA can be applied for treating larger tumors. The single antenna MWA reduces the number of punctures required, leading to a decreased incidence of complications. Currently, the most commonly used image-guided technologies including ultrasound, CT, and MRI [16], of which ultrasound guidance is most widely used due to the advantage of real-time monitoring and simple operation [5–8]. For liver cancer near the diaphragm, especially near the top of the diaphragm, the ultrasounds works poorly, and sometimes cannot display the tumor due to the impact of pulmonary gas and ribs [9–12]. Note that, in our study a 68-year-old male received ultrasound-guided MWA for liver S8 cancer with a diameter of 1.2 cm in another hospital. A 1-month follow-up showed that the lesions were still located in the original position, and a low-density ablation lesion appeared adjacent to the original liver cancer (Figure 1). A 57-year-old female underwent ultrasound-guided MWA for a liver S8 metastatic tumor with a diameter of 1.8 cm. The post-operative review showed that a small portion of the lesion was ablated (Figure 2). These two cases had a history of an “off-target” phenomenon after ultrasound-guided MWA outside our hospital. CT-guided MWAs were successfully implemented in our hospital, and the lateral review showed that the liver tumors were completely ablated.

Clear displays of tumor and puncture path are the most important conditions for MWA. With the advent of artificial pleural effusion [17] and artificial ascites [18], ultrasound-guided MWA for liver tumors near the diaphragm has become possible [19–21]. Song et al. [20] retrospectively analyzed 143 patients with 148 HCC lesions with a diameter < 4 cm near the diaphragm, and the CR rate was 85.3%, which was significantly lower than our study. Park et al. [21] retrospectively analyzed 116 liver tumors with diameters < 3 cm, of which 56 were near the diaphragm. The CR rate was 87.5%, which was significantly lower than our study, and even lower than the 99.0% in the group of liver tumor diameters with a < 3 cm. Nam et al. [22] analyzed 28 HCC lesions with diameters < 5 cm near the diaphragm, and the LR rate of the artificial ascites-assisted (n=15) and control groups (n=13) was 26.7% and 23.1%, which were both higher than our study. With respect to ultrasound-guided WMA for an artificial pleural effusion, Koda et al. [19] retrospectively analyzed 23 patients with HCC lesions with a diameter < 5 cm near the top of diaphragm and the CR rate was 88.0%, which was significantly lower than our study. Zhang et al. [23] retrospectively analyzed 31 HCC lesions with diameters < 5 cm near the diaphragm, and the LR rate was 16.1%, which was significantly higher than our study. Regarding the treatment for liver tumors near the diaphragm, CT-guided MWA through the lung had a higher CR rate and lower LR rate compared to ultrasound-guided MWA with artificial ascites or artificial pleural effusions.

Through group study, we showed that the efficacy of CT-guided MWA through the lung for liver tumors near the diaphragm was impacted by the diameter of the liver tumor. The efficacy of Group I (diameter ≤ 3.0 cm) was significantly better than Group II (diameter > 3.0 cm). The CR rate of Groups I and II were 99.0% (100/101) and 80.0% (24/30), respectively (*χ2*=16.525, *P*=0.001). The result was similar to the Head et al. [24] study of RFA for liver tumors near the diaphragm in which they found that the local progression of tumors > 3 cm in diameter was greater than tumors < 3 cm (70.6% vs. 12.5%). The following reasons explain why liver tumors > 3 cm in diameter have worse therapeutic efficacy. Indeed, it is difficult to achieve complete ablation with a one-time single antenna; multiple adjustments of the antenna are required for ablation, and it is difficult to set a reasonable ablation power and time to avoid residual tumor.

For the 117 patients with 131 liver tumors near the diaphragm in the current study, there were no fatal and disabling complications after CT-guided MWA through the lung. The total complication rate was 23.6%, which was slightly higher than that reported for ultrasound-guided MWA [19–22]. This finding may be accounted for as follows: the puncture procedure cannot be shown in real-time under CT guidance, and it is heavily impacted by patient breathing activity. CT guidance cannot monitor the real-time ablation range of an ablation antenna. Of note, we could take several measures to reduce the complication rate. First, teach patients to do breathing exercises before MWA. Second, evaluate the path of blood vessels around the tumor and along the puncture path before MWA; contrast-enhanced CT is recommended when necessary. Third, a low power, an extended ablation time, an intense monitor, and a shortened scan interval are suggested to be applied for large liver tumors near the diaphragm. Fourth, pay attention to monitoring diaphragmatic thickening and pulmonary exudative changes. All of the complications in our study were minor. There were no complaints of significant discomfort and no treatment needed, and the patients were discharged after a 2-day intense observation period. Moreover, the most severe complications of WMA were injury to the neighboring dangerous organs [25–30]. In agreement with the study involving ultrasound-guided MWA with artificial ascites or artificial pleural effusions for liver tumors near the diaphragm [19–22], no severe complications occurred in our study. In subgroup analysis, the incidence of pneumothoraces was 15.3%, which was significantly lower than the 45% (5/11) in the RFA study conducted by Shibata et al., the 37.5% (9/24) in the Kato et al. study [31], the 71.4% (5/7) in the Toyoda et al. study, [32] and the 45% (17/38) in the Park et al. study [33]. CT-guided MWA through the lung is a safe method, but the complication rate is significantly impacted by the size of liver tumors. The complication rate for liver tumors with a diameter > 3.0 cm was significantly greater than liver tumors ≤ 3.0 cm in diameter (43.3% vs. 19.8%; [*χ2*=6.796], *P*=0.015). All of the severe complications occurred in patients with liver tumors > 3.0 cm in diameter. For liver tumors with a relatively large diameter, multiple adjustments of the antenna are needed during MWA, which may lead to an increase in the rate of complications, such as the incidence of pneumothoraces in patients with liver tumors > 3.0 cm in diameter compared to liver tumors ≤ 3.0 cm in diameter (26.7% and 11.9%, respectively; [*χ2*=3.909], *P*=0.048). Several studies have reported that the incidence of pneumothoraces is associated with the length of the lung puncture path, number of punctures, emphysema, and other factors [24, 25]. Longer ablation time and higher power were required to achieve complete ablation for larger tumors, which could also increase the ablation range and ablation-related complications.

Compared to the ultrasound-guided MWA assisted artificial pleural effusion or ascites for liver tumors near the diaphragm, CT-guided MWA had several advantages. First, CT guidance has a higher ablation success rate, with high repeatability. The success of ultrasound-guided MWA for liver tumors near the diaphragm depends on the establishment of an artificial pleural effusion or ascites; however, the technology of artificial pleural effusion or ascites relies heavily on the operator’s experience and cannot ensure successful implementation for all patients, thus it would impact the next step of ablation. Rahim et al. [34], Song et al. [20] and Park et al. [22] reported that the success rate of artificial ascites was 88%, 90.9%, and 86.7%, respectively. Lin et al. [35] and Zhang et al. [23] reported that the success rate of artificial pleural effusion was 95.0% and 98.2%, respectively. Moreover, for patients who underwent artificial pleural effusion or suffered from pleurisy and patients who underwent surgical resection of liver tumors, transarterial chemoembolization, hepatic tumor ablation, or had peritonitis, it is more difficult to establish artificial pleural effusion or ascites due to the peritoneal or pleural adhesions and normal anatomic position changes [20, 23, 34]. Therefore, ultrasound-guided artificial ascites and artificial pleural effusion-assisted MWA for liver tumors near the diaphragm are difficult to repeat. Second, CT-guided MWA can clearly judge the ablation range, the degree of thickening of the diaphragm, and the leaking change of lung tissues near the diaphragm, therefore CT-guided MWA can directly determine the degree of damage to the diaphragm. During MWA, the thickening or coarse diaphragm indicates that the edge of the ablated thermal coagulation necrosis was close to the diaphragm, which would cause stress-induced edema of the diaphragm. An intra-operative CT scan that shows the right lower lung has a flake- or flocculent-shape dense shadow suggests that the heat generated during MWA passed through the diaphragm to the lung, leading to exudative changes. When the above phenomenon occurs, the ablation should be promptly stopped to avoid damage to the diaphragm. There were 22 patients with diaphragm thickening and 8 patients with pulmonary exudative changes, but none of the patients had significant complications after MWA. Due to interference of the gas generated in the process of liver tumor coagulation necrosis by ablation, ultrasound-guidance cannot clearly display the ablation range, the diaphragm, and the ablation reaction of lung tissue near the diaphragm. Third, all of the complications caused by CT-guided MWA through the lung are controllable and treatable. It took 1-2 weeks for patients to completely absorb the pleural effusion and ascites after the artificial pleural effusion and artificial ascites were established. Moreover, artificial pleural effusion requires chest tube placement for persistent drainage [19, 34], resulting in a greater impact on the treatment and life of patients.

There were some limitations to this study. First, the study was a single-center regression analysis; a multi-center, multi-case, and long-term follow-up to evaluate the efficacy and safety of CT-guided MWA through the lung for liver tumors near the diaphragm is warranted. Second, selection bias was possible because there was a large difference in the number of patients between the group with intra-hepatic tumor diameters < 3 cm and the group with tumor diameters ≥ 3 cm. Despite the limitations, these results may contribute to guiding the MWA ablation treatment of liver tumors near the diaphragm.

## MATERIALS AND METHODS

### Patients

A retrospective study was conducted involving 131 liver tumors that were close to the diaphragm in 117 patients who underwent CT-guided MWA between January 2011 and July 2014. The study was approved by the Institutional Review Board of Sun Yat-sen University Cancer Center. Written informed consent was obtained from all patients before therapy. The patient inclusion criteria consisted of the following: (1) single hepatic tumor with a diameter ≤ 6 cm, or multiple hepatic tumors with the largest diameter ≤ 3 cm and tumor number ≤ 3; (2) good general condition, Karnofsky scale (KPS) ≥70; Eastern Cooperative Oncology Group Performance Status score: 0-1; (3) class A or B Child-Pugh score for liver function; and (4) a platelet count > 50×10^9^/L, a neutrophil count > 2×10^9^/L, an international normalized thrombin time ratio > 1.5, and a haemoglobin (HGB) ≥ 100 g/L. Of the 117 patients, there were 91 males and 26 females; the average age was 57.4 ±11.4 years (range, 25-79 years). One hundred two patients had single liver tumors close to the diaphragm, 13 patients had 2 liver tumors close to the diaphragm, and 1 patient had 3 liver tumors close to the diaphragm. The average tumor diameter was 2.27±1.14 cm (range, 0.6-6.0 cm). Forty-eight liver metastases and 83 primary liver tumors were included. All patients with liver metastases were pathologically-confirmed, and the primary tumors were all under control by surgery. The diagnosis for patients with primary liver tumors all met American Association for the Study of Liver Diseases (AASLD) criteria. The tumors were sorted based on size. The tumors were sorted to Group I if the size of the tumor was < 3.0 cm (n=101 tumors) and the tumors were sorted to Group II if the size of the tumor was ≥ 3.0 cm (n=30 tumors). The baseline information of patients was shown in Table 2.

**Table 2:** The baseline information of all the patients

Variables	Group I	Group II	*P* values
	(n=101)	(n=30)	
**Sex (n)**			0.289
female	17	8	
male	84	22	
**Age (year)**	56.2±11.76	59.0±10.32	0.647
**Size (cm)**	1.76±0.61	3.96±0.81	0.254
**Diagnose**			0.051
HCC	69	14	
Metastases	32	16	

Values with “±” are written as mean ± SD; HCC: hepatocellular carcinoma.

The tumors were sorted to Group I if the size of the tumor was < 3.0 cm and the tumors were sorted to Group II if the size of the tumor was ≥ 3.0 cm.

### Microwave ablation

All patients underwent contrast-enhanced CT or MRI scan 2 weeks before surgery to determine the size and location of the tumors. During treatment, CT was used to locate the liver tumors (PHILIPS 16-slice spiral CT [90-cm pore size and 5-mm scanning thickness]; The Netherlands) and to design the optimal puncture needle route. Routine disinfection and local anaesthesia was applied around the puncture point, and a 16G microwave antenna was inserted gradually into the tumor along the pre-determined angle. The entire MWA procedure treatment was conducted with intravenous anaesthesia (propofol [1.5∼4.5 mg/kg continuous intravenous infusion/h]; AstraZeneca S.p.A., Italy) and under real-time ECG monitoring. The setting of the MWA parameters depended on the manufacturer’s recommendation and our experience. The ablation area must completely cover the tumor and exceed the tumor edge to approximately 0.5 cm. Chest and upper abdominal CT scans were obtained out immediately post-operatively to evaluate whether or not the ablation area was sufficient, and whether or not there were complications, such as bleeding and a pneumothorax. Routine blood testing, biochemical testing, and chest X-rays were carried out the first day after MWA treatment. If the related examination results showed no abnormalities and the patient was recovering satisfactorily, the patient was discharged the following day. One hundred seventeen patients with 131 liver tumors close to the diaphragm all successfully underwent CT-guided MWA through the lung.

MWA equipment: A microwave delivery system (FORSEA; Qinghai Microwave Electronic Institute, Nanjing, China) was used in the studies. This system consisted of a MTC-3 microwave generator (FORSEA) with a frequency of 2450 MHz, a power output of 10-150 W, a flexible low-loss cable, and a 16-gauge cooled-shaft antenna.

### Follow-ups

Upper abdominal contrast-enhanced CT or MRI scans were carried out every month for the first 3 months post-operatively. If no tumor residue or tumor recurrence was detected, a re-examination was carried out every 3-6 months (CT: HiSpeed or LightSpeed QX/i, GE Medical Systems; Milwaukee, WI; contrast agent: Ultravist 300 [injection speed was fixed as 3ml/s]; Schering, Berlin, Germany; MRI: Discovery MR750 3.0T; GE Medical Systems; contrast agent: Gd-DTPA [injection speed was fixed to 3ml/s]; GE Healthcare, IDA Business Park Carrigtohill, Ireland).

### Therapeutic efficacy and complication assessment

#### Therapeutic efficacy evaluation

(1) A complete response (CR; Figure 1) was defined as tumor ablation area that did not show enhanced lesions on the follow-up liver CT/MRI scan. (2) An incomplete response (ICR) or “tumor residue” was defined as a residual enhanced lesion in the tumor ablation area on the follow-up liver CT/MRI scan. (3) A local recurrence (LR) was defined as a CR on the first month follow-up CT/MRI scan, but an enhanced area inside or at the edge of the ablation area on subsequent review.

#### Complications

Severe complications included complications which appeared within 2 weeks following MWA, and if no treatments were given to patients, could cause prolonged hospitalization, an increased disability rate, or death. All other complications were defined as mild.

It is worth noting that congestive edema of the diaphragm and mild right lung exudative change during the MWA treatment process are the only stress response to MWA, and were not considered to be complications. Congestive edema of the diaphragm was defined as diaphragmatic thickening or coarse-like changes on the CT obtained immediately after ablation. A mild right lung exudative change was defined as a flake- or flocculent-shape dense shadow in the lung.

### Statistical analysis

SPSS for OS X (version 20.0; SPSS, Inc., Chicago, IL, USA) was used for statistical analysis. Intergroup *t*-tests were used for quantitative data, and intergroup *χ2* tests were used for count data. A *P* <0.05 was considered statistically significant.

## CONCLUSIONS

CT-guided MWA through the lung for treating liver tumors has the advantages of clear imaging, accurate positioning, and accurate puncture into the liver tumors near the diaphragm. CT-guided MWA can clearly monitor the changes in the diaphragm and the lung tissues near the diaphragm during the ablation process. CT-guided MWA can also make up some of the defects of ultrasound-guided MWA with good therapeutic efficacy. Furthermore, all of the complications that occurred during ablation are treatable. Thus, CT-guided MWA through the lung plays an important role in treating liver tumors near the diaphragm.

## Abbreviations

MWA: microwave ablation
CR: complete response
ICR: incomplete response
LR: local recurrence
KPS: Karnofsky Scal
